# Exploring Smart Furniture: A Systematic Review of Integrated Technologies, Functionalities, and Applications

**DOI:** 10.3390/s25226900

**Published:** 2025-11-12

**Authors:** Inês Mimoso, Marcelo Brites-Pereira, Leovaldo Alcântara, Maria Inês Morgado, Gualter Morgado, Inês Saavedra, Francisco José Melero Muñoz, Juliana Louceiro, Elísio Costa

**Affiliations:** 1 RISE—Health, Competence Center for Active and Healthy Ageing, Faculty of Pharmacy, University of Porto, Rua Jorge de Viterbo Ferreira 228, 4050-313 Porto, Portugal; ifmimoso@ff.up.pt (I.M.); up202001410@edu.icbas.up.pt (L.A.); mimorgado@ff.up.pt (M.I.M.); 2 Competence Center for Active and Healthy Ageing, Faculty of Pharmacy, University of Porto, Rua Jorge de Viterbo Ferreira 228, 4050-313 Porto, Portugal; marcelobritespereira@gmail.com; 3 APIMA—Associação Portuguesa da Indústria de Mobiliário e Afins, Rua da Constituição 395, 4200-199 Porto, Portugal; gualter.morgado@apima.pt; 4 SHINE 2Europe, Rua Câmara Pestana, Lote 3—1°D/F, 3030-163 Coimbra, Portugal; inessaavedra@shine2.eu (I.S.); julianalouceiro@shine2.eu (J.L.); 5 Technological Research Centre of Furniture and Wood of the Region of Murcia, 30510 Murcia, Spain; fj.melero@cetem.es

**Keywords:** smart furniture, IoT, sensor networks, health monitoring, ambient assisted living, human-centred design

## Abstract

**Highlights:**

**What are the main findings?**

- Identifies three core technological pillars in smart furniture—data collection (sensors), transmission/processing (IoT), and actuation—with health monitoring as the dominant application.
- Reveals a pre-commercial gap; 37% of prototypes were validated only in laboratory settings, 20% exclusively through user testing, and just 23% underwent both types of validation. Only one study achieved TRL 9 and is already commercialised.

**What are the implications of the main findings?**

- Urges participatory design: Highlights the need for co-creation with end-users to bridge the gap between prototypes and market-ready solutions.
- Calls for standardisation: Emphasises ethical data governance and interoperable frameworks to enable scalable, context-aware smart furniture systems.

**Abstract:**

Smart furniture represents a growing field that integrates Internet of Things (IoT), embedded systems and assistive technologies, yet lacks a comprehensive synthesis of its components and applications. This PRISMA-guided systematic review analysed 35 studies published between 2014 and 2024, sourced from PubMed, Web of Science and Scopus. The included studies presented prototypes of smart furniture that used IoT, sensors or automation. The focus was on extracting data related to technological configurations, functional uses, validation methods, maturity levels and commercialisation. Three technological pillars emerged, data collection (*n* = 31 studies), transmission/processing (*n* = 30), and actuation (*n* = 22), often combined into multifunctional systems (*n* = 14). Health monitoring was the dominant application (*n* = 15), followed by environmental control (*n* = 8) and assistive functions for older adults (*n* = 8). Validation methods varied; 37% relied solely on laboratory testing, while 20% only involved end-users. Only one solution surpassed Technology Readiness Level (TRL) 7 and is currently on the market. Current research remains pre-commercial, with gaps in AI integration, long-term validation, and participatory design. Smart furniture shows promise for healthcare and independent living, but requires standardised evaluation, ethical data practices, and co-creation to achieve market readiness.

## 1. Introduction

In recent decades, technology has revolutionised the way of life and the range of choices, especially with the emergence of autonomous and semi-intelligent devices [1]. Therefore, furniture needs to keep pace with changes in user expectations, becoming smarter and more interactive to suit our modern spaces [1,2]. The combination of the Internet of Things (IoT), environmental intelligence and Human-Centred design (HCD) is transforming furniture from simple objects into dynamic and interactive systems.

With the advent of Industry 4.0, advanced digital technologies such as the IoT are being integrated into manufacturing, creating environments where devices, appliances and users can communicate effortlessly [3]. These innovations are becoming a staple in smart homes, where everyday items are equipped with sensors, remote controls and intelligent systems. In this perspective, smart furniture is defined as “furniture designed and networked, equipped with an intelligent system or controlled by the user’s data and energy sources” [4]. This type of furniture utilises sensors and actuators to interact with users and anticipate their needs, all to improve quality of life in a connected and intelligent world [5,6].

The ageing of the global population is also an important factor driving the development of smart furniture [7]. As people age, they often face physical and cognitive challenges that can make everyday tasks more difficult and increase the risk of injury, especially falls [8]. Smart furniture can play a crucial role in helping older people maintain their independence, improve their quality of life and encourage active and healthy ageing by addressing some of the challenges that accompany this demographic shift [7]. Instead of relying on specialised or stigmatising products aimed solely at older people, it is possible to incorporate assistive features into ordinary furniture following universal design principles. This approach enhances everyday usability without drawing attention to age-related limitations.

Smart furniture can be effectively leveraged to support active and healthy ageing not only in domestic environments, but also in professional and public contexts. Several studies [9] have demonstrated that ICT solutions, such as sensors, actuators, reminders, and activity tracking systems, integrated into furniture can enhance autonomy, well-being, and participation among older adults. These systems are most effective when designed with the user’s specific needs and contexts in mind. In both home and workplace settings, smart furniture has demonstrated utility in promoting cognitive stimulation, improving postural health, and reducing risks related to physical inactivity or falls. This underscores the importance of developing adaptable, context-aware solutions that respond to the realities of ageing populations across different life domains, moving beyond the home and into the broader ecosystem of daily life.

Although there is growing interest in technology-enhanced furniture that makes everyday life easier, development in this area faces some difficult challenges. A major obstacle is the lack of standardised methods to guide the process from design to implementation, which leads to fragmented solutions that often lack consistent usability, safety, and interoperability [4]. Although approaches such as HCD are suggested, their use in industrial contexts is still quite limited and slow to be adopted [10]. Furthermore, the addition of data-driven functionalities raises important questions about privacy and cybersecurity, especially when untrustworthy stakeholders are part of the development process.

As interest in smart furniture continues to grow, there is still a notable gap in comprehensive analyses that thoroughly examine the technologies, functionalities, and applications of these innovations. This systematic review aims to fill that gap by addressing the following questions: What types of integrated technologies were applied to smart furniture prototypes between 2014 and 2024, and what were their functions? How were they evaluated in terms of development maturity, validation and commercialisation? The review compiles the current state of research on smart furniture, providing insights into this evolving field by mapping existing technologies and their uses. In doing so, it hopes to serve as a valuable resource for researchers, designers, and industry professionals.

In this review, “technology” refers to embedded physical components or digital systems integrated into furniture, designed to (1) collect data (e.g., sensors); (2) process/transmit information (e.g., microcontrollers, networks); or (3) execute physical/digital actions (e.g., actuators, robotics). Software algorithms or non-electronic materials are excluded unless directly implemented in hardware.

This review makes key contributions to the field of smart furniture and sensor-based systems. First, it offers a comprehensive synthesis of technological configurations across 35 prototypes, identifying three core pillars, data collection, transmission/processing, and actuation, and mapping their integration into multifunctional systems. Second, it introduces an assessment of TRLs, highlighting the pre-commercial status of most solutions and the methodological gaps that hinder scalability. Third, it draws attention to the lack of participatory design, underscoring the need for inclusive and standardised development frameworks. By bridging technical analysis with implementation challenges, this review provides a valuable resource for researchers, designers, and industry stakeholders aiming to advance smart furniture from concept to market.

The paper is structured as follows: Section 2 outlines the methodology, Section 3 presents the findings, Section 4 discusses the implications, and Section 5 concludes with recommendations for future work.

## 2. Materials and Methods

### 2.1. Study Design

This systematic review followed the model proposed by the Joanna Briggs Institute, PRISMA (Preferred Reporting Items for Systematic Reviews and Meta-Analyses) strategy for systematic literature reviews [11]. Researchers first defined the scope of the study and established the eligibility criteria, with a focus on integrating technologies in smart furniture. While not prospectively registered in PROSPERO, the full protocol (including search strings and data extraction forms) is available in Appendix A.

### 2.2. Data Sources and Search Strategy

A comprehensive search was conducted in PubMed, Web of Science (WoS), and Scopus covering the period from January 2014 to December 2024. The search string included terms such as “smart furniture”, “IoT”, “Ambient Assisted Living”, and “health monitoring”, combined with Boolean operators. The search strategy for each database is provided in Appendix A.1 (Table A1).

### 2.3. Eligibility Criteria

The eligibility criteria for this systematic review were established to include studies specifically focused on the integration of technologies into smart furniture. The aim was to identify the types of technologies employed, their functional roles, and their level of technological maturity. Studies were selected based on their relevance to the development and validation of smart furniture prototypes, with particular attention to applications in health, well-being, and ambient assisted living. The exclusion of non-English articles was defined a priori to ensure consistency in data extraction and avoid potential translation bias. To enhance transparency and reproducibility, the inclusion and exclusion criteria are presented in Table 1.

Studies published between 2014 and 2024 were prioritised due to the technological maturity of IoT and the relevance of application contexts (e.g., smart cities and homes). Conference papers were included to ensure the representation of practical, albeit preliminary, solutions, thereby complementing more in-depth analyses from journal articles.

### 2.4. Article Search and Selection

**Figure 1:**
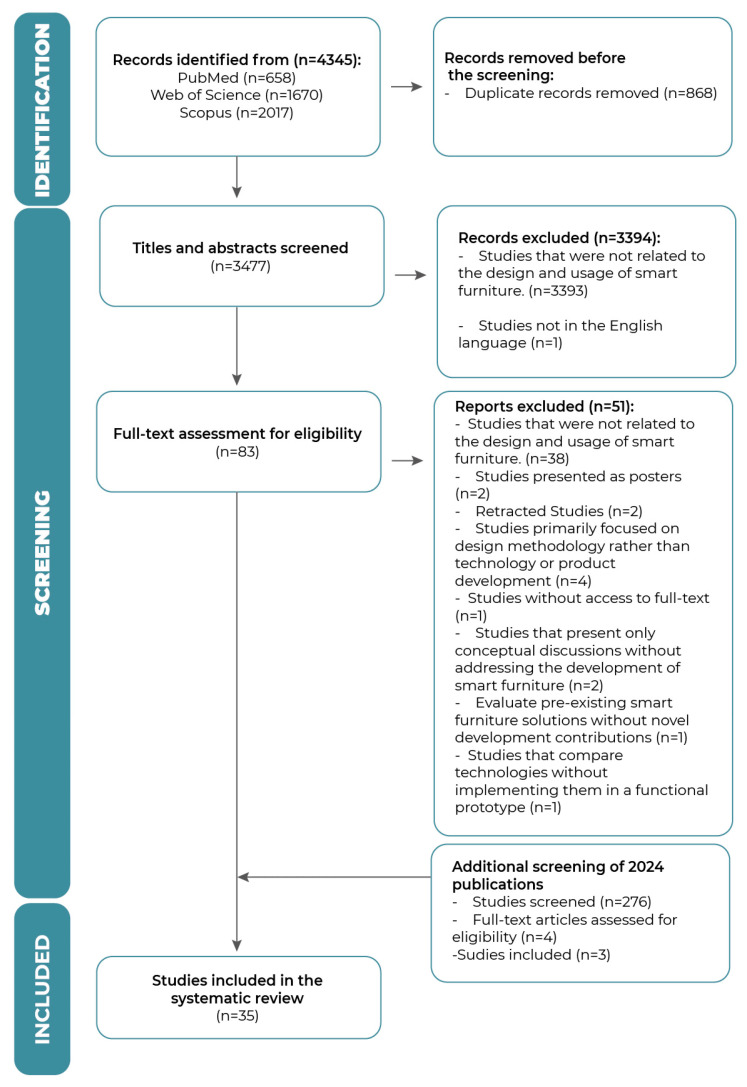
PRISMA flowchart of the study inclusion and exclusion process.

After searching the databases, the articles found were exported to the Rayyan software [12], where duplicates were removed. The articles were then analysed (Rayyan AI was used in blind screening mode) by titles, abstracts, and full text by two reviewers, IM and MB, who applied the inclusion and exclusion criteria. Any disagreements between the two reviewers were resolved by a third reviewer, EC. The entire selection process is illustrated in a PRISMA flowchart (see Figure 1).

### 2.5. Data Extraction and Synthesis

Data were extracted using a standardised form, which included the following categories: Type of publication (e.g., journal article, conference paper); Year; Region/Country where the study was conducted; Technology Used; Technology Function; Furniture Typology; Smart Furniture Functions; Type of Data Collected; Validation Process; Technology Readiness Levels (TRL); Information about Commercialisation. Furthermore, additional data related to technologies or creation processes were also analysed and integrated into the discussions. A narrative synthesis was performed to summarise the findings, focusing on trends, patterns, and gaps in the literature. Where applicable, quantitative data were analysed using descriptive statistics, and results were presented in tables and figures to facilitate comparison across studies.

### 2.6. Validity Assessment

To control the reliability and validity of the 35 [13,14,15,16,17,18,19,20,21,22,23,24,25,26,27,28,29,30,31,32,33,34,35,36,37,38,39,40,41,42,43,44,45,46,47] selected studies, the technological level of each solution was analysed using the TRL scale (NASA/EC standards). As this is applied research on smart furniture, the studies included were evaluated based on their stage of development, rigour of validation and applicability in the real world. For each study, the degree of maturity was classified using the following levels: TRL Level 7–9 (High Maturity): Systems that have been validated in an operational (industrial) environment/tested by end users themselves (large-scale prototypes, demonstrators). TRL 4–6 (Moderate Maturity): Prototypes tested in laboratories or other controlled environments and some field tests (e.g., user studies, some field trials). TRL 1–3 (Low Maturity): Conceptual designs, calculations, or proof of concept in the laboratory. To avoid the risk of bias, two authors (IM and MB) individually assigned a TRL for inclusion in the high TRL forecast, based on how the validation had been reported (experimentally, user testing, or scalability). Differences were discussed with a third author (LA) using the methods and results of the original study.

### 2.7. Use of Generative Artificial Intelligence

During the preparation of this manuscript, we employed generative AI tools (ChatGPT-4 and DeepSeek-V3.2) to assist with three key aspects of our research: initial text generation for certain sections, refining our study design through iterative questioning about potential improvements, and exploring data correlations in the systematic review. Importantly, all AI-generated suggestions underwent rigorous human evaluation and were substantially reworked by the authors to ensure accuracy and alignment with our standards. The final manuscript represents our original academic work, with AI serving solely as a supplemental tool for ideation and analysis refinement.

## 3. Results

In the selection process, a total of 4621 records were found in the Web of Science, PubMed, and Scopus databases, covering the years 2014 to 2024. This period was chosen to ensure that both the most recent advances and previous contributions in the field could be captured. Following the PRISMA guidelines for systematic reviews, two researchers (IM and MB) independently carried out the screening process. In the second phase, 87 full articles were evaluated for eligibility. After applying the inclusion and exclusion criteria, 52 studies were excluded, resulting in a final selection of 35 articles for data extraction and synthesis (see Table A2, Appendix A.2).

### 3.1. Study Characteristics

The studies analysed came primarily from conference papers (*n* = 21) [22,23,24,25,26,27,28,29,30,33,34,35,36,37,38,39,40,41,42,43,44], while a smaller number were journal articles (*n* = 14) [13,14,15,16,17,18,19,20,21,31,32,45,46,47]. These papers were published between 2014 and 2024, with a notable spike in activity in 2020 when (*n* = 7) [14,18,29,30,31,32,33] studies were published.

**Figure 2:**
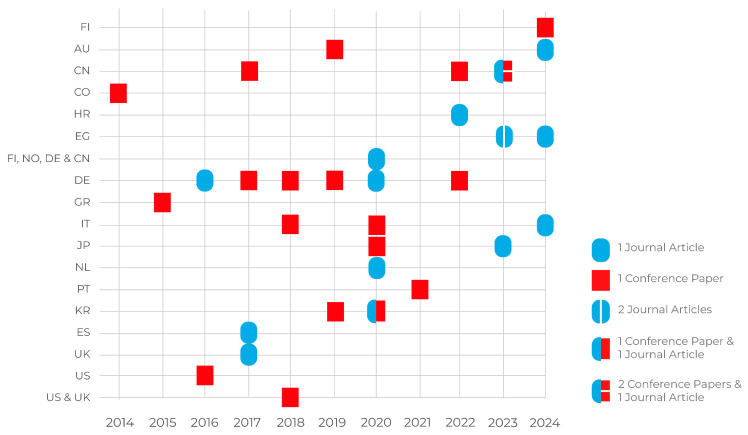
Study characteristics: year, country and type of publication.

Geographically, the research was conducted primarily in Germany (*n* = 6) [16,18,26,35,40,42], followed by China (*n* = 5) [19,23,24,27,38], the Republic of Korea (*n* = 3) [30,31,37], Italy (*n* = 3) [20,33,39], Egypt (*n* = 3) [21,46,47], Japan (*n* = 2) [13,29], Australia (*n* = 2) [34,45], and a few other countries that contributed one study each, Colombia [43], Croatia [25], Greece [44], the Netherlands [14], Portugal [28], Spain [17], the United Kingdom [15], and the United States. We also found two international collaborations: one between researchers from the UK and the US [36], and another involving teams from Finland, Norway, Germany, and China [32], as seen in Figure 2.

**Figure 3:**
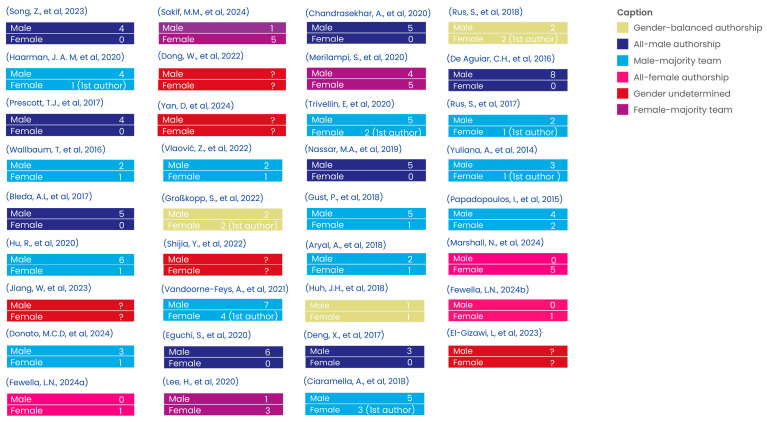
Study characteristics: Gender and authorship [13,14,15,16,17,18,19,20,21,22,23,24,25,26,27,28,29,30,31,32,33,34,35,36,37,38,39,40,41,42,43,44,45,46,47].

When analysed closely, the authorship patterns across the 35 studies revealed some significant gender imbalances in who was contributing to smart furniture research. As observed in Figure 3., the majority of authorship teams were male-dominated (*n* = 21) [13,14,15,16,17,18,20,25,28,29,31,33,34,35,37,38,40,41,42,43], with eight studies [13,15,17,29,31,33,37,40] written entirely by men. On the other hand, gender-balanced teams (*n* = 3) [26,36,39] and those with a majority of female authors (*n* = 3) [22,30,32] were quite rare. Only two studies [21,46] had a female independent author. There was only one study [45] in which the group of authors consisted exclusively of women. This trend continued when we examined first authors, where male first authors (*n* = 18) [13,15,16,17,18,20,22,25,29,30,31,32,34,35,36,38,41,44] outnumbered female first authors (*n* = 12) [14,21,24,26,28,33,39,40,42,43,45,46] by almost two to one. In five cases [19,23,24,27,47], we were unable to determine the gender of all the authors due to unclear names or missing information.

### 3.2. Technologies Identified in Smart Furniture

The review of the 35 studies included in this analysis revealed a diverse range of technological components that are part of smart furniture systems, listed here by frequency of occurrence. The most commonly used were Arduino microcontrollers and Raspberry Pi single-board computers, closely followed by load cells, RFID/NFC systems, and Bluetooth modules. A variety of environmental sensors were also prevalent, covering aspects such as temperature, humidity, air quality, light, and moisture levels.

Furthermore, various types of force and pressure sensors (such as FSR, capacitive sensors, and load cells), along with motion and proximity sensors (including ultrasonic, PIR, infrared, and radar), were frequently mentioned. Other common components included Zigbee and WiFi wireless modules, electrocardiogram (ECG) sensors, photoplethysmographic (PPG) sensors, thermistors, and optical fibre sensors.

Actuation and feedback technologies were also part of the mix, featuring servo motors, linear actuators, LED matrices, LCD displays, and voice recognition modules (like Baidu Voice Assistant and the LD3320 chip). Additionally, some studies integrated Artificial Intelligence and Machine Learning elements, such as convolutional neural networks, decision trees, random forests, support vector machines (SVM), and Naive Bayes algorithms. Cloud computing platforms and robotic components—like mobile bases and robotic arms—were also utilised.

On a less frequent note, some studies made use of triboelectric nanogenerators (TENGs), Leap Motion controllers, Xbee RF modules, and programmable logic controllers (PLCs). A handful of technologies were only mentioned in single studies, including April tags, Kinect motion sensors (Microsoft Corporation, WA, USA), thermal cameras, reactive-ion etching (RIE) systems, Hetai HT66F2390 microcontrollers (Holtek Semiconductor Inc., Hsinchu, Taiwan), ATK-AS608 fingerprint modules, HC-SR501 human body sensors, M38 Bluetooth audio receivers, 5W power amplifier modules, and specialised deep learning chips for voice recognition.

To provide a clearer overview of the technological configurations and their application domains, Table 2 presents a structured summary of the furniture types, integrated technologies, and their functional roles across the selected studies.

### 3.3. Technological Roles

**Figure 4:**
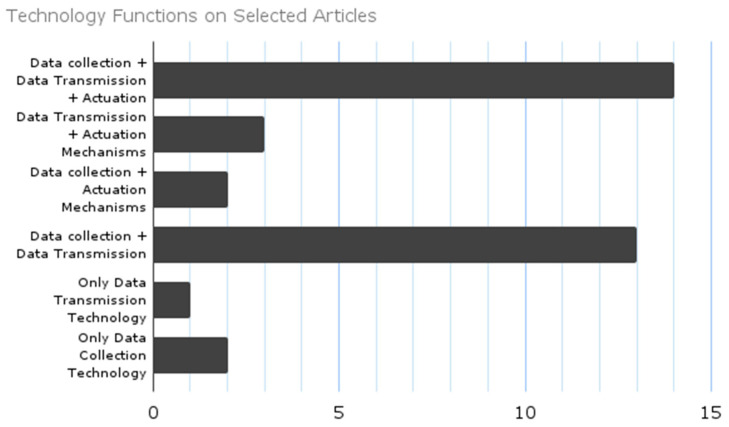
Distribution of technology functions across selected articles. Three core categories of technology functions were assessed: sensor systems for data collection, data transmission and processing technologies, and actuation mechanisms enabling physical and/or digital outputs.

The analysis revealed three key categories of technological components that were integrated across the studies: sensor systems for gathering data (*n* = 31) [13,14,15,16,17,18,19,21,22,23,24,25,26,30,31,32,33,34,35,36,37,38,39,40,41,42,43,44,45,46,47] technologies for data transmission and processing (*n* = 30) [14,16,17,18,20,21,22,23,24,25,26,27,28,29,30,31,32,33,34,36,37,38,39,40,41,42,43,45,46,47], and actuation mechanisms that handle physical and/or digital outputs (*n* = 22) [14,15,16,17,22,23,24,25,26,27,28,29,30,31,35,36,37,43,44,45,46,47]. When it comes to how these components were put together, the studies showed a variety of configurations. Specifically, we discovered: Studies that utilised only data collection technologies (*n* = 2) [13,19]; Studies that focused solely on data transmission/processing technologies (*n* = 1) [20]; Studies that combined data collection with transmission/processing technologies (*n* = 13) [15,18,21,32,33,34,38,39,40,42,45,46,47]; Studies that merged data collection with actuation mechanisms (*n* = 2) [35,44]; Studies that integrated transmission/processing technologies with actuation mechanisms (*n* = 3) [27,28,29]; Studies featuring fully integrated systems that included all three categories of technologies (*n* = 14) [14,16,17,22,23,24,25,26,30,31,36,37,41,43] (see Figure 4). This classification not only underscores the popularity of sensor-based approaches but also points to a growing trend towards multifunctional, interconnected smart furniture systems.

### 3.4. Furniture Types and Application Domains

The studies analysed showed a variety of furniture types that have been adapted for smart functionalities. This included a range of items such as tables (*n* = 7) [14,15,16,22,26,43,47] sofas (*n* = 5) [19,23,24,40,42] chairs (*n* = 3) [29,33,43], mirrors (*n* = 4) [21,32,38,46], desks (*n* = 3) [28,31,36], outdoor furniture (*n* = 4) [33,34,39,45], bedside tables (*n* = 2) [17,27], beds (*n* = 2) [17,18], office chairs (*n* = 2) [25,35], and cabinets (*n* = 2) [30,37]. There were also unique pieces like armchairs (*n* = 1) [17], kitchen cabinets and dining furniture (*n* = 2) [18,41], drawer units (*n* = 2) [21,46], and main entrance pieces (*n* = 1) [44]. On top of that, two studies [13,20] introduced modular technologies that can be added to existing furniture, allowing it to gain smart features without needing entirely new smart furniture. Throughout these studies, the smart furniture systems were crafted to fulfil six main functional roles through their built-in technologies. The most prevalent application was health and well-being monitoring (*n* = 15) [14,17,18,19,21,22,23,24,25,32,35,36,40,42,47], which included features like posture correction and tracking physiological parameters. Environmental monitoring, which covers activities such as tracking ambient conditions and detecting earthquakes, was noted in (*n* = 8) studies [13,19,20,33,34,38,39,45]. Functions aimed at assisting specific age groups, such as anticipating dangerous situations or encouraging physical activity, also appeared in (*n* = 8) studies [15,17,18,22,24,32,41,47]. Enhancing social interaction, especially through non-verbal communication, was another focus in (*n* = 7) studies [14,16,21,22,23,26,45]. Workspace adaptability, such as desks with reconfigurable height, was highlighted in (*n* = 5) systems [25,28,35,36,38]. Lastly, tools for improving efficiency and managing daily life, including organisational support, were integrated into (*n* = 11) smart furniture solutions [15,27,29,30,31,37,38,41,43,44,46].

### 3.5. Technology Validation

The studies showed quite a bit of variation in how they validated their findings, and we can break them down into three main categories. The most popular method was experimental testing, with (*n* = 13) studies relying solely on this method [13,14,19,20,22,28,29,31,34,35,39,46,47], which usually took place in labs. These studies focused on validating prototypes by checking their structural integrity, conducting load tests, and evaluating technical performance, like how accurate the sensors were and how responsive the systems were under controlled conditions.

Only seven studies (*n* = 7) [24,26,32,40,41,42,45] relied solely on user validation, conducting pilot studies with end-users, Wizard-of-Oz (WOZ) techniques, scenario-based focus groups, and post-deployment surveys to gauge usability and relevance in real-life situations. Interestingly, (*n* = 8) studies [16,17,21,25,27,30,33,36] used a hybrid validation framework, blending the thoroughness of technical testing with user-focused assessments. This combination allowed for a more rounded evaluation by merging lab-based analyses with feedback from current users. However, there was a significant gap, (*n* = 7) [15,18,23,37,38,43,44] studies did not clearly outline their validation processes, which makes it harder to assess the transparency and reproducibility of their results.

### 3.6. Technology Maturity Assessment

The assessment of the TRL of the studies considered in this systematic review showed an interesting picture of the development phase of the smart furniture solutions investigated. The distribution of TRLs illustrated that most technologies (85,7%) are at intermediate TRL levels (TRL 4–6)—functional prototypes tested in controlled or laboratory environments, without having been extensively tested in real applications. Two studies [18,20] reached TRL 7, proving to be in the pre-commercial phase, having established industrial collaborations. No studies were conducted at TRL 8, and only one study [45] reached TRL 9, highlighting a significant communication gap between the bench scale and the product on the market. The analysis of application categories revealed that those related to health and well-being, especially for older adults, show greater technological maturity (average TRL 6). On the other hand, more innovative applications, such as emotional smart furniture and future home automation, show lower maturity (average TRL 4). It is also interesting that most of the projects with higher TRL (≥6) have important methodological aspects in common: (1) iterative design; (2) validation with end users; and (3) integration with existing technological platforms (e.g., IoT, digital health systems). All these factors seem to contribute strongly to accelerating the maturity of the solutions.

Table 3 summarises the publication type, country of origin, validation method, TRL classification, and commercialisation status of each study, offering a comparative view of technological maturity and implementation potential.

### 3.7. Commercialisation

The systematic review revealed that the vast majority of studies (*n* = 34) focused exclusively on prototypes or developments in the conceptual phase. Only one study [45] evaluated a commercially available product. Although a small subset of studies (*n* = 5) [18,20,21,38,39] mentioned possible commercialisation pathways (registered patents or discussed business plans), these provided insufficient detail on actual market implementation, regulatory approvals, or production scaling. This pattern suggests that the field remains predominantly in the transition phase from research to commercialisation, with limited evidence of real-world implementation.

## 4. Discussion

This systematic review brings together a full decade of research on smart furniture, highlighting that the field is still in its experimental phase. This can be seen in the fact that there are more conference papers (20 of 35) than journal articles (15 of 35), a trend often observed in rapidly changing technology fields [4,48]. While conference proceedings do not always have the same level of methodological rigour, they allow for the rapid sharing of ideas in areas where technology can quickly become obsolete. Many of the studies focus on prototype innovations, but only a few make it past the proof-of-concept phase—often referred to as the “valley of death” in innovation [49].

The peak in publications occurred in 2020 (*n* = 7), probably influenced by the COVID-19 pandemic, which changed research priorities and intensified the production of manuscripts in several areas [50]. However, it is not yet known whether the pandemic changed the focus or depth of these studies. When the geographic distribution is examined, it is clear that high-income regions dominate the area—Europe (*n* = 17) and Asia (*n* = 10) lead, while only a few studies came from South America (Colombia), Africa (Egypt), Oceania (Australia) or North America (USA). This disparity likely points to differences in funding and infrastructure for technology research [51,52]. Expanding the scope of the databases or including grey literature could help improve representation.

Our analysis revealed a significant gender imbalance in authorship, with male-dominated teams contributing to the majority of studies (*n* = 21). This disparity aligns with broader trends in STEM fields [53] but has specific implications for smart furniture research. A lack of gender diversity in design teams can inadvertently lead to biased technological development, where products may not adequately address the needs or preferences of all end-users. For instance, solutions aimed at older adults, a demographic with a high proportion of women, may overlook key ergonomic, aesthetic, or usability concerns if female perspectives are underrepresented in the design process. Furthermore, our reliance on inferred gender data due to the absence of self-reporting highlights a need for more transparent demographic reporting in academic publications. Moving forward, fostering inclusive and participatory design practices is not only an ethical imperative but also a crucial step towards creating smart furniture that is truly adaptable and responsive to a diverse user base [54]. These findings are not merely descriptive; they highlight structural gaps in research practices that may influence the inclusivity, relevance, and scalability of smart furniture solutions. By examining geographic and gender-related patterns, this review aims to promote more equitable innovation and encourage future studies to adopt inclusive design frameworks that reflect diverse user needs and contexts.

### 4.1. Trends and Technological Priorities

The field relies on three key technological components: sensor systems (*n* = 31) [13,14,15,16,17,18,19,21,22,23,24,25,26,30,31,32,33,34,35,36,37,38,39,40,41,42,43,44,45,46,47], data transmission and processing (*n* = 30) [14,16,17,18,20,21,22,23,24,25,26,27,28,29,30,31,32,33,34,36,37,38,39,40,41,42,43,45,46,47] and actuation mechanisms (*n* = 22) [14,15,16,17,22,23,24,25,26,27,28,29,30,31,35,36,37,43,44,45,46,47]. Many studies are combining these elements, indicating a shift away from autonomous technologies toward more interconnected systems. Sensor systems gather environmental and biometric data, while transmission components facilitate real-time decision-making, and actuators enable responsive interactions. This integration of technologies can enhance interoperability but also risks premature standardisation, which may hinder disruptive innovation [53].

When it comes to data collection, the focus has been on environmental factors (such as temperature and light), biometric information (such as heart rate and posture), and interaction inputs (such as gestures and voice). This supports applications in health monitoring (*n* = 15) [14,17,18,19,21,22,23,24,25,32,35,36,40,42,47], efficiency management (*n* = 11) [14,17,18,19,21,22,23,24,25,32,35,36,40,42,47], and environmental control (*n* = 8) studies [13,19,20,33,34,38,39,45]. However, there is a notable lack of applications targeting social interaction or age-specific needs (both *n* = 8) studies [15,17,18,22,24,32,41,47], pointing to a bias towards individual performance over collective or assistive use cases [55,56].

While continuous biometric data collection is highly dependent, most studies overlook key issues such as privacy, security, and regulatory standards (like General Data Protection Regulation (GDPR) and Food and Drug Administration (FDA)), raising concerns about data governance and the reliability of these technologies as medical devices [57]. Another significant observation is the trend towards retrofitting: smart features are often added to traditional furniture rather than inspiring entirely new designs. This approach can limit innovation and lead to ergonomic issues (such as increased bulk due to embedded sensors), missing opportunities for more integrated, human-centric solutions like those seen in wearables [58].

### 4.2. Design, Use Cases, and Contextual Integration

Smart furniture is moving from being just basic structures to becoming interactive elements, both inside our homes (think adaptive chairs) and in public areas (such as solar-powered benches). Unfortunately, the lack of standardised evaluation methods results in a lot of variability between different studies, making comparisons and replication of results difficult [59].

None of the studies has mentioned co-creation or participatory design. In cases where users have been included, their involvement occurs at later stages of development, thereby limiting the potential impact of their contributions. The use of participatory approaches is particularly relevant in contexts involving older people, where usability, accessibility, and user needs must be prioritised from the outset [60,61]. Such approaches can also help prevent the development of unnecessary technologies, which are often driven more by technical feasibility or commercial interests than by clearly defined social or health needs [62]. Furthermore, none of the reviewed studies targeting older adults, a demographic with specific health and usability needs, explicitly reference global policy frameworks such as the WHO’s call for inclusive technology to support healthy ageing [63]. This omission suggests a disconnect between technological development and internationally recognised guidelines, potentially limiting the relevance, scalability, and ethical robustness of these solutions.

Furthermore, the concentration of studies in Europe assumes that technological infrastructures (such as broadband and reliable electricity) are available everywhere, which is not the case. Solutions that rely on constant connectivity or cloud-based AI are unlikely to work well in resource-poor environments [64]. Future research should focus on how to adapt to different contexts, including the use of edge computing and modular designs that can be globally relevant.

### 4.3. Gaps in Validation and Path to Market

The analysis of validation methods across studies reveals a disjointed methodological approach, shaped primarily by three main trends. First, laboratory testing alone dominates (*n* = 13) [13,14,19,20,22,28,29,31,34,35,39,46,47], with most studies confined to controlled experiments. Second, human-centred validation remains surprisingly scarce: seven studies [24,26,32,40,41,42,45] rely exclusively on user testing, while eight others [16,17,21,25,27,30,33,36] combine user and experimental testing, yet often overlook critical factors like ergonomic fit and accessibility. Finally, empirical validation is alarmingly limited (*n* = 7) [15,18,23,37,38,43,44], undermining both reproducibility and real-world applicability. These shortcomings are closely linked to Technology Readiness Level assessments: 5.7% of solutions don’t surpass TRL1–3 (low maturity), 85.7% of solutions stagnate at TRL 4–6 (moderate maturity), while only 8.6% advance to TRL 7–9 (high maturity). Importantly, the limited presence of TRL 9 solutions is not due to selection bias in our review process, but rather reflects the current state of the field. In response to the research question, it is noteworthy that the studies with higher TRLs did not employ mixed-method validation approaches. Rather, they relied solely on either user testing or experimental procedures. This observation suggests that as smart furniture solutions advance in maturity, validation practices may become more streamlined or one-dimensional [65,66]. These findings highlight a notable gap in the validation frameworks applied to smart furniture, indicating a lack of consistency or comprehensiveness in how such technologies are assessed across studies.

This highlights a persistent tendency to focus on technical feasibility, often to the detriment of human factors, which echoes criticisms found in the smart furniture literature [67]. Such an imbalance risks creating systems that do not align with real user needs, even if they perform well in lab environments [68]. For example, healthcare applications designed for older people users reached TRL between 4–6, but hit a roadblock in the pre-commercial stages (TRL 7) due to unresolved issues with scalability and regulatory compliance. Notably, the review found only one commercially available product among 35 studies, despite two patent applications, highlighting a widespread “valley of death” between prototypes and market-ready solutions—a gap compounded by insufficient validation [49]. It is also important to acknowledge that some studies may report only a portion of the validation process, omitting prior stages or contextual details. Such omissions can weaken the reader’s understanding of the overall evaluation framework and obscure the continuity between development and validation phases.

The limited commercial success in this field appears closely linked to these research practices. Broader adoption challenges, including high costs, privacy concerns, and usability hurdles for non-technical users [69], are further complicated by the lack of focus on real-world constraints. On the other hand, the transition from research to commercialisation faces a structural barrier: the disconnect between SMEs and research centres [70,71]. While prototypes achieve TRL 6–7 in controlled environments, their scalability depends heavily on innovation hubs that translate academic solutions into market-ready products.

### 4.4. Challenges and Future Directions

This review highlights significant challenges faced when in developing and implementing smart furniture, especially for health and wellness applications. Key issues include concerns about data privacy and security, particularly in health monitoring scenarios; the need to integrate technology without compromising aesthetics, which has not yet been sufficiently explored; and the limited involvement of end users in many studies, which may hinder adoption and real-world relevance. To address these challenges, future research should: (1) Adopt participatory design frameworks that involve end users throughout the development process, ensuring that smart furniture genuinely meets their needs, preferences, and ethical standards. (2) Conduct longitudinal validation studies to assess the reliability, durability, and accuracy of sensors over time and across various usage conditions. (3) Investigate culturally adaptable and accessible interaction methods to foster inclusion across different age groups, cognitive abilities, and physical capabilities. (4) Establish clearer reporting standards for technology validation and user evaluation processes. (5) Develop sustainable business models that support the scalability and long-term maintenance of smart furniture systems. (6) Address ethical issues more directly, especially concerning data ownership, informed consent, and surveillance in data-sensitive applications. Thus, highlighting the importance of embedding participatory design approaches throughout the development of smart furniture solutions, particularly when targeting older adults or vulnerable users. Co-creation processes involving end-users can surface privacy concerns early and improve emotional acceptance, usability, and trust in technology. Evidence shows that when older adults are engaged in iterative design cycles, their perceived usefulness and willingness to adopt smart solutions increases substantially. Therefore, participatory methodologies should be considered a foundational element in future developments, ensuring that ethical principles are upheld not only in data management but in the very architecture of smart environments.

By tackling these gaps, future research can help develop smart furniture solutions that are ethically sound, contextually appropriate, and widely adopted [72].

### 4.5. Limitations of the Review

The inclusion of conference proceedings allowed us to capture emerging innovations and cutting-edge prototypes. However, this may have introduced some variability in the quality of methodologies and depth of reporting, which could impact the overall reliability of certain findings. Despite this limitation, we considered it crucial to include these sources to accurately map the current landscape of this rapidly evolving field. This review did not include patents to maintain methodological integrity. Patent documents often miss the mark when it comes to providing solid empirical data on usability, validation, or human-centric outcomes [73]. While they can shed light on early-stage technological innovations, they often promote unverified commercial claims that can distort systematic synthesis. Looking ahead, future scoping reviews or additional analyses could truly benefit from using patent databases to track innovation trends that go beyond the peer-reviewed literature.

## 5. Conclusions

This systematic review took a close look at 35 studies published between 2014 and 2024, providing a thorough analysis of the technologies, functionalities, and applications found in smart furniture. The findings show that this field is rapidly evolving, fuelled by technological advancements and a growing responsiveness to user needs, especially in areas related to health and well-being. On the tech side, sensor-based systems were commonly used for tasks like environmental monitoring, biometric data collection and user interaction. Embedded computing platforms are also widely used, with Arduino and Raspberry Pi standing out as popular options. However, despite these advancements, the limited use of artificial intelligence indicates that the field has not fully tapped into predictive and adaptive capabilities, focusing more on data collection than on intelligent responses. The review also points out some exciting applications for ageing populations, such as fall-detection systems and posture-monitoring chairs. Yet, it notes that only seven studies have clear paths to commercialisation, highlighting a significant gap between academic innovation and practical implementation. Geographically, the concentration of studies in Europe raises some concerns about how well these findings can be applied to other regions with different infrastructures and cultural contexts. Methodologically, the fact that 60% of the studies come from conference proceedings reflects the experimental and emerging nature of this field, but it also signals a need for more rigorous, peer-reviewed validation. From this review, three key priorities stand out: 1. Long-term, real-world testing is crucial for moving from lab prototypes to commercially viable products. 2. The need to standardise evaluation frameworks to ensure that solutions can be compared, replicated, and refined based on solid evidence. 3. Human-centred, cross-disciplinary design should steer development by weaving together insights from engineering, design, health sciences, and urbanism. This approach ensures that everything created is not only functional and aesthetically pleasing but also ethically sound.

For smart furniture to grow sustainably, it needs to adopt universal design principles, encourage interoperability among IoT systems, and explore open-source or patent-free models, thereby fostering ecological and inclusive innovation.

## Figures and Tables

**Table 1:** Eligibility Criteria for Study Inclusion and Exclusion.

Criteria Type	Inclusion Criteria	Exclusion Criteria
Publication Date	Articles published between 2014 and 2024	Articles published before 2014 or after 2024
Language	Written in English	Written in other languages
Study Format	Journal articles and conference papers	Posters, notes, abstracts, patents, or grey literature.
Technological Focus	Furniture with embedded smart systems, IoT devices, AAL technologies, or domotics	Studies focused on materials, logistics, adhesives, or unrelated industrial aspects
Application Context	Health monitoring, smart home technologies, and urban smart furniture	Studies without prototype development or with purely conceptual discussions
Prototype Development	Describes the development and/or validation of a smart furniture prototype/system	Studies that only compare technologies or evaluate existing solutions without novelty
Availability	Full-text available	Only the abstract is available or inaccessible

**Table 2:** Reference, Type of Furniture, Type of Technology, Application.

Reference	Furniture Type	Integrated Technology	Technology Role	Application
[13]	Can be built into existing furniture such as desk, etc	Ultrahigh-frequency-band radiofrequency identification (RFID)	Data collection	Environment monitoring
[14]	Table	Load cell HX711 24-Bit Analogue-to-Digital Converter (ADC) LED	Data collection, Process/transmit information, Execute physical/digital actions	Social interaction and communication, Health and well-being
[15]	Table	APRIL tags Robotic components	Data collection, Execute physical/digital actions	Targeted to older people, Efficiency and Daily Life Management
[16]	Table	Raspberry Pi LED Arduino Mega RFID-tags proximity sensor	Data collection, Process/transmit information, Execute physical/digital actions	Social interaction and communication
[17]	Bed, an armchair and a bedside table	Wireless Sensor Networks (WSN) Zigbee modules Microcontrollers Wireless node Load cells Accelerometers Moisture sensor Magnetic contact sensor Infrared temperature Infrared distance sensors Motor actuators Presence sensor Light sensor	Data collection, Process/transmit information, Execute physical/digital actions	Targeted to older people, Health and well-being
[18]	interactive kitchen & dining furniture bed	Electrocardiogram (ECG) sensors Pressure-sensing mattresses Kinect motion sensors thermal cameras. Cloud-based platform AI-based predictive analytics. (IoT)	Data collection, Process/transmit information	Health and well-being, Targeted to older people
[19]	Sofa	Optical fibre sensor	Data collection	Environment monitoring, Health and well-being
[20]	Can be built into existing furniture such as desk, etc	Internet of Things LoRa, FSK, UWB cloud computing	Process/transmit information	Environment monitoring
[21]	Wall-mounted mirror and a drawer unit	Motion Sensors: Ultrasonic sensors Microcontroller: Arduino	Data collection, Process/transmit information	Social interaction and communication, Health and well-being
[22]	Table	IFTTT (If This Then That) Cloud-Based Web Interface, NFC tags	Data collection, Process/transmit information, Execute physical/digital actions	Targeted to older people, Social interaction and communication, Health and well-being
[23]	Sofa	Artificial Intelligence (AI) IoT Bluetooth Artificial Neural Networks (ANN) algorithm temperature and sound sensors	Data collection, Process/transmit information, Execute physical/digital actions	Social interaction and communication, Health and well-being
[24]	Sofa	Deep Learning Chip for Voice Recognition Sensors for blood pressure, heart rate, body temperature, and blood oxygen monitoring. GPRS Communication Mechanical Actuators for Electric Motors and Heating Elements	Data collection, Process/transmit information, Execute physical/digital actions	Health and well-being, Targeted to older people
[25]	Office chairs	Force-sensitive resistors (FSR), capacitive sensors, photoplethysmographic (PPG) electrocardiographic (ECG) radar sensors, and flex sensors connected to IoT Microcontrollers Arduino Raspberry Pi ESPino32.	Data collection, Process/transmit information, Execute physical/digital actions	Health and well-being, Work/office spaces
[26]	Table	Projector Camera and Object Recognition Smartphone Integration LED Matrix recorder	Data collection, Process/transmit information, Execute physical/digital actions	Social interaction and communication
[27]	Bedside Table	Microcontroller LCD Display Fingerprint module adopts Recognition module Human body sensing Bluetooth audio receiver modulePower amplifier module	Process/transmit information, Execute physical/digital actions	Efficiency and Daily Life Management
[28]	Desk	Lifting mechanisms (electric, crank, pin slide) LED light sensor Arduino Relay	Process/transmit information, Execute physical/digital actions	Work/office spaces
[29]	Chair	Six-axis sensor Ultrasonic sensor (HC-SR04)	Process/transmit information, Execute physical/digital actions	Efficiency and Daily Life Management
[30]	Storage Cabinets	Infrared communication sensors and servo motors. Arduino UNO Voice commands	Data collection, Process/transmit information, Execute physical/digital actions	Efficiency and Daily Life Management
[31]	Desk	Triboelectric Nanogenerator (TENG) Arduino Uno Reactive-Ion Etching (RIE).	Data collection, Process/transmit information, Execute physical/digital actions	Efficiency and Daily Life Management
[32]	2 Chairs 1 Mirror	Infrared sensorscamera and facial recognition module Bluetooth pressure sensors (Biometrics Ltd., UK)	Data collection, Process/transmit information	Health and well-being, Targeted to older people
[33]	Outdoor furniture (e.g., benches, vases, fountains, street furniture)	Cloud platform Sensors: temperature, humidity, air quality, atmospheric pressure, brightness, and soil moisture.	Data collection, Process/transmit information	Environment monitoring
[34]	Outdoor furniture—bins, seats, bus shelters	Wireless sensor nodes (Arduino Uno R3 (Arduino, Ivrea, Italy), ESP-13 WiFi shield (Espressif Systems, Xangai, China)) ultrasonic and temperature sensors	Data collection, Process/transmit information	Environment monitoring
[35]	Office chairs	Capacitive sensors microcontrollers Bluetooth actuators	Data collection, Execute physical/digital actions	Health and well-being, Work/office spaces
[36]	Desk	IoT sensors Raspberry Pi RFID tag Power over Ethernet Machine learning algorithms	Data collection, Process/transmit information, Execute physical/digital actions	Health and well-being, Work/office spaces
[37]	shoe cabinet	Raspberry Pi Arduino UNO Pressure sensors Convolutional Neural Network (CNN) IoT, Deep Learning (AI)	Data collection, Process/transmit information, Execute physical/digital actions	Efficiency and Daily Life Management
[38]	Mirror	Raspberry Pi, TFT, LCD display, infrared frame, Baidu Voice Assistant	Data collection, Process/transmit information	Environment monitoring, Efficiency and Daily Life Management, Social interaction and communication
[39]	Smart urban furnishings (benches, worktops, digital islands)	Sensors RFID WiFi NFC	Data collection, Process/transmit information	Environment monitoring
[40]	Sofa	Sensorsmachine learning algorithms	Data collection, Process/transmit information	Health and well-being
[41]	Robotic furniture suite (chair, side table, and screen).	Sensors Modules, Leap Motion controller Arduino Stepper motors Linear actuators LED	Execute physical/digital actions, Data collection, Process/transmit information	Targeted to older people, Efficiency and Daily Life Management
[42]	Sofa	Textile capacitive sensing electrodesmicrocontroller	Data collection, Process/transmit information	Health and well-being
[43]	Chairs and tables	Sensors Raspberry Pi Zigbee wireless	Data collection, Process/transmit information, Execute physical/digital actions	Efficiency and Daily Life Management
[44]	Main entrance furniture piece	Weight Sensors PLC (Programmable Logic Controller)	Data collection, Execute physical/digital actions	Efficiency and Daily Life Management
[45]	Hub: Street Furniture	Sensors Wi-Fi IoT	Data collection, Process/transmit information, Execute physical/digital actions	Environment monitoring Social interaction and communication
[46]	Mirror and Drawer	IoT, Sensors	Data collection, Process/transmit information, Execute physical/digital actions	Efficiency and Daily Life Management
[47]	Office furniture	Sensors Ergonomic mechanisms Modular systems	Data collection, Process/transmit information, Execute physical/digital actions	Work/Office spaces

**Table 3:** Reference, Publication Type, Country, Year, Validation, TRL, Commercialisation.

Reference	Publication Type	Country	Year	Validation	TRL	Commercialisation
[13]	Journal Article	Japan	2019	Experimental Test	6—Tested in controlled environment, but with real data	No
[14]	Journal Article	Netherlands	2019	Experimental Test	5—Validated in the laboratory with feeding behaviour tests.	No
[15]	Journal Article	United Kingdom	2024	Not Specified	4—Prototype without testing in real scenarios.	No
[16]	Journal Article	Germany	2014	Experimental + User Study	5—Tested in a controlled environment with users.	No
[17]	Journal Article	Spain	2022	Experimental + User Study	6—Tested in real environment, but on a small scale	No
[18]	Journal Article	Germany	2020	Not Specified	7—Pilot phase with industrial partners (pre-commercialisation).	Future iterations aim for market deployment through European industries and healthcare providers
[19]	Journal Article	China	2020	Experimental Test	4—Validated only in the laboratory.	No
[20]	Journal Article	Italy	2017	Experimental Test	7—In operational implementation phase	Ongoing implementation in the VITALITY project; aims for TRL 7 (real operational environment) and future commercialization.
[21]	Journal Article	Egypt	2018	Experimental + User Study	5—Tested with users (ADHD), but not on a large scale.	Is in the process of being patented, with potential for commercialization in the Egyptian market and beyond.
[22]	Conference Paper	Finland	2024	Experimental Test	4—Functional prototype, without large-scale testing.	No
[23]	Conference Paper	China	2023	Not Specified	3—Without physical prototype or validation	No
[24]	Conference Paper	China	2020	User Study	4—Limited user testing (no real environment).	No
[25]	Journal Article	Croatia	2019	Experimental + User Study	6—Tested in real environment (office).	No
[26]	Conference Paper	Germany	2017	User Study	5—Tested in 3 homes (real scenario, but small sample).	No
[27]	Conference Paper	China	2020	Experimental + User Study	4—Basic functionalities validated in the laboratory.	No
[28]	Conference Paper	Portugal	2016	Experimental Test	6—Ergonomics tests on prototype	No
[29]	Conference Paper	Japan	2023	Experimental Test	4—Without testing in real use	No
[30]	Conference Paper	Republic of Korea	2020	Experimental + User Study	5—Tested with users (WOZ technique).	No
[31]	Journal Article	Republic of Korea	2021	Experimental Test	5—Validated in laboratory and simulations (without external testing).	No
[32]	Journal Article	Finland, Norway, Germany and China	2016	User Study	6—Tested in usability studies.	No
[33]	Conference Paper	Italy	2023	Experimental + User Study	4—Tested in simulated scenarios.	No
[34]	Conference Paper	Australia	2023	Experimental Test	5—Tested in an urban environment (small scale).	No
[35]	Conference Paper	Germany	2024	Experimental Test	6—Tested with 12 users (empirical data).	No
[36]	Conference Paper	USA and UK	2020	Experimental + User Study	6—Iterative development (versions 1–3 with user feedback).	No
[37]	Conference Paper	Republic of Korea	2018	Not Specified	3—Conceptual prototype without validation.	No
[38]	Conference Paper	China	2020	Not Specified	4—Patent pending (no large-scale testing).	The project is pending patent applications, but no explicit commercialisation details are provided in the text.
[39]	Conference Paper	Italy	2017	Experimental Test	5—Tested in academia-industry collaboration.	The project aims to define a sustainable business plan, but no explicit commercialisation details are provided in the text.
[40]	Conference Paper	Germany	2018	User Study	5—Tested with 15 participants.	No
[41]	Conference Paper	USA	2022	Experimental + User Study	4—Initial usability testing.	No
[42]	Conference Paper	Germany	2017	User Study	5—Validated with 15 users (cross-validation).	No
[43]	Conference Paper	Colombia	2015	Not Specified	4—Prototype without testing in a real environment.	No
[44]	Conference Paper	Greece	2022	Not Specified	5—Market plan, but without implementation	No
[45]	Journal Article	Australia	2024	User Study	TRL 9—Actual system proven in operational environment	Product already commercialised
[46]	Journal Article	Egypt	2024	User Study	4—Prototype without testing in real scenarios.	No
[47]	Journal Article	Egypt	2023	User testing	6—Prototype demonstrated in relevant environment	No

## Data Availability

The original contributions presented in this study are included in the article. Further inquiries can be directed to the corresponding author.

## Abbreviations

The following abbreviations are used in this manuscript:

IoT	Internet of Things
TRL	Technology Readiness Level
HCD	Human-centred Design
PRISMA	Preferred Reporting Items for Systematic Reviews and Meta-Analyses
WoS	Web Of Science
AAL	Ambient Assisted Living
ECG	Electrocardiogram
PPG	Photoplethysmography
SVM	Support Vector Machine
TENG	Triboelectric Nanogenerators
PLCs	Programmable Logic Controllers
RIE	Reactive-ion etching
WOZ	Wizard of Oz
GDPR	General Data Protection Regulation
FDA	Food and Drug Administration

## Appendix A

### Appendix A.1

**Table A1:** Search strings and database yields used in the systematic review. Boolean operators (OR/AND) were applied to combine terms across PubMed, Web of Science, and Scopus (total initial results: n = 4621).

Database (Results)	Search String
PubMed	(“Smart furniture”[TIAB] OR (IoT[TIAB] AND furniture[TIAB]) OR (“Ambient assisted living”[TIAB] AND furniture[TIAB]) OR (Domotics[TIAB] AND furniture[TIAB]) OR (“Smart Homes”[TIAB] AND furniture[TIAB]) OR (technology[TIAB] AND furniture[TIAB]) OR (“Assistive technology”[TIAB] AND furniture[TIAB]) OR (Ergonomic[TIAB] AND furniture[TIAB] AND technology[TIAB]) OR (furniture[TIAB] AND “health monitoring”[TIAB]) OR (“Ambient intelligence”[TIAB] AND furniture[TIAB]))
Web of Science	“Smart furniture” OR (IoT AND furniture) OR (“Ambient assisted living” AND furniture) OR (Domotics AND furniture) OR (“Smart Homes” AND furniture) OR (technology AND furniture) OR (“Assistive technology” AND furniture) OR (Ergonomic AND furniture AND technology) OR (Furniture AND “health monitoring”) OR (“Ambient intelligence” AND furniture)
Scopus	TITLE-ABS-KEY (“Smart furniture” OR (“IoT” AND furniture) OR (“Ambient assisted living” AND furniture) OR (domotics AND furniture) OR (“Smart Home” AND furniture) OR (technology AND furniture) OR (“Assistive technology” AND furniture) OR (ergonomic AND furniture AND technology) OR (furniture AND “health monitoring”) OR (“Ambient intelligence” AND furniture)
Total	n = 4621

### Appendix A.2

**Table A2:** Characteristics of the 32 included studies, categorised by type of publication, evidence level, country of publication, technology used, furniture typology (sensors, data transmission, actuation), functional application, data collection, validation method and commercialisation.

Reference	Year	Type of Pub.	Region/Country	Integrated Technology	Technology Role	Furniture Typology	Smart Furniture Functions	Notes on Smart Furniture Functions	Type of Data Collected	Validation Process	Technology Readiness Levels (TRL)	Info. About Commercialization?
[13]	2019	Journal Article	Japan	Ultrahigh-frequency-band radiofrequency identification (RFID)	Data collection	Can be built into existing furniture such as desk, etc	Environment monitoring	Monitoring eathquakes	Observational vibration results from a long-term monitoring earthquakes	Describes the process to validate the system’s functionalities: vibrations from earthquakes (experimental testing)	6—Tested in controlled environment, but with real data	No
[14]	2019	Journal Article	Netherlands	Load cell HX711 24-Bit Analogue-to-Digital Converter (ADC) LED	Data collection, Process/transmit information, Execute physical/digital actions	Table	Social interaction and communication, Health and well-being	Sensory Interactive Table that allows to study the eating behaviours and social interaction of people in a social setting	Eating Behaviour Detection: Amount of food consumed by individuals. Monitors water intake. Identifies which foods are eaten first or left untouched. Detects the arrangement of dining items (plates, glasses, pots) on the dining table. Behavioural analysis during meals. Monitoring food and water consumption patterns.	Describes the process to validate the system’s functionalities: Load cells to measures pressure. (experimental testing)	5—Validated in the laboratory with feeding behaviour tests.	No
[15]	2024	Journal Article	United Kingdom	APRIL tags Robotic components	Data collection, Execute physical/digital actions	Table	Targeted to older people, Efficiency and Daily Life Management	Cantilever table able to move around by itself, or under user control.	Movement validation metrics Orientation data Relative spatial coordinates	No	4—Prototype without testing in real scenarios.	No
[16]	2014	Journal Article	Germany	Raspberry Pi LED Arduino Mega RFID-tags proximity sensor	Data collection, Process/transmit information, Execute physical/digital actions	Table	Social interaction and communication	Table that promotes non-verbal communication between people living over a distance.	User Identification Data Event-Driven Interaction Data	Laboratory observation with usability tasks and questionnaires. lab study in a controlled environment. (experimental testing and user testing)	5—Tested in a controlled environment with users.	No
[17]	2022	Journal Article	Spain	Wireless Sensor Networks (WSN) Zigbee modules Microcontrollers Wireless node (AT86RF230) Load cells (LAA-W1) 3-axis accelerometers—ADXL345BCCZ-RL Moisture sensor Magnetic contact sensor Infrared temperature sensors (MLX90614)—temperature sensor Infrared distance sensors (GP2Y0A21YK0F) Passive infra-red (PIR) sensors- presence sensor Light sensor (APDS-9007-020) LED Thermistor (NTCLE100E3103JB0)—temperature sensor Motor actuators	Data collection, Process/transmit information, Execute physical/digital actions	Bed, an armchair and a bedside table	Targeted to older people, Health and well-being	Smart Sensory Furniture that anticipates human needs, inferring a potentially dangerous action of an elderly person living alone at home.	Motion and Presence Detection Monitor user proximity, detect fever anomalies, and infer presence. Detect liquid spills (e.g., urine, water) for hygiene alerts. Measure weight distribution; detect user presence/activity (e.g., falls). Detect furniture tilt/impact (fall detection), monitor bed positioning. Monitor room activity; trigger assistive lighting. Adjust ambient lighting automatically (e.g., nighttime safety). Enable real-time data transmission between nodes and base station.	Two stages process validation: Off-Furniture Tests (Lab Testing); In-Furniture Tests (Real-World Testing) (experimental testing and user testing)	6—Tested in real environment, but on a small scale	No
[18]	2020	Journal Article	Germany	Electrocardiogram (ECG) sensors Pressure-sensing mattresses Kinect motion sensors thermal cameras. Cloud-based platform AI-based predictive analytics. Internet of Things (IoT)	Data collection, Process/transmit information	interactive kitchen & dining furniture bed	Health and well-being, Targeted to older people	Furniture that encourages activity and customised healthcare in the context of an ageing society. Monitors health, cognitive and physical activation.	ECG signals, respiration rate, temperature, pressure data Gesture tracking, stand-up counting, Room conditions, light levels, and object positioning		7—Pilot phase with industrial partners (pre-commercialisation).	Future iterations aim for market deployment through European industries and healthcare providers
[19]	2020	Journal Article	China	Optical fibre sensor	Data collection	Sofa	Environment monitoring, Health and well-being	A multifunctional embedded optical fibre system for furniture, enabling light guidance and environmental or user interaction monitoring	Temperature, humidity, and carbon dioxide levels. Detection of sitting duration and stress on the furniture. Monitoring of structural integrity and stress on the 3D-printed components.	Several experiments: Temperature Sensing Experiment; Structural Strength Experiment (experimental testing)	4—Validated only in the laboratory.	No
[20]	2017	Journal Article	Italy	Internet of Things (IoT) LoRa, FSK, UWB cloud computing	Process/transmit information	Can be built into existing furniture such as desk, etc	Environment monitoring	Detects earthquakes, signals the presence of individuals and supports rescue operations	Presence detection, environmental monitoring, acceleration data for earthquake detection.	Validated through prototype testing, laboratory experiments, simulations, and UAV-assisted localization tests. (experimental testing)	7—In operational implementation phase	Ongoing implementation in the VITALITY project; aims for TRL 7 (real operational environment) and future commercialization.
[21]	2018	Journal Article	Egypt	Motion Sensors: Ultrasonic sensors Microcontroller: Arduino	Data collection, Process/transmit information	Wall-mounted mirror and a drawer unit	Social interaction and communication, Health and well-being	The technologies were chosen to create an interactive piece of furniture that promotes family integration, especially for children with ADHD	User Presence and Height, Behavioural Responses interacted with the furniture and how the lighting responses influenced their behaviour, and user feedback	Prototype Testing using technical testing; User Testing (experimental testing and user testing)	5—Tested with users (ADHD), but not on a large scale.	Is in the process of being patented, with potential for commercialization in the Egyptian market and beyond.
[22]	2024	Conference Paper	Finland	IFTTT (If This Then That) Cloud-Based Web Interface, NFC tags	Data collection, Process/transmit information, Execute physical/digital actions	Table	Targeted to older people, Social interaction and communication, Health and well-being	Accessible smart mobile devices available for people with motor or cognitive difficulties (older people, patients with dementia, etc.). They can be used in home or assisted living environments to promote autonomy and simple interaction.	User Interaction, system performance and environmental Data.	The system was validated through functionality testing and practical evaluation. (experimental testing)	4—Functional prototype, without large-scale testing.	No
[23]	2023	Conference Paper	China	Artificial Intelligence (AI), Internet of Things (IoT), Bluetooth, Artificial Neural Networks (ANN) algorithm, temperature and sound sensors	Data collection, Process/transmit information, Execute physical/digital actions	Sofa	Social interaction and communication, Health and well-being	Light adjustment, wireless charging, Bluetooth connectivity, mood-based lighting, temperature and sound sensing, entertainment integration integrated in a smart sofa for young people living alone.	User mood, behaviour, sound, temperature, and lighting preferences	No	3—Without physical prototype or validation	No
[24]	2020	Conference Paper	China	Deep Learning, Chip for Voice Recognition (LD3320) and sensors for blood pressure, heart rate, body temperature, and blood oxygen monitoring. GPRS Communication, Mechanical Actuators for Electric Motors and Heating Elements	Data collection, Process/transmit information, Execute physical/digital actions	Sofa	Health and well-being, Targeted to older people	Health Monitoring, Voice Control, Massage Function, Heating Function, Childcare Function, Stand-Up Assistance and Autonomous Learning. Integrated into a cognitive smart sofa that uses advanced technologies for autonomous interaction with the user.	Blood pressure, heart rate, body temperature, and blood oxygen levels are collected and stored in the cloud. User Preferences, voice Data for authentication and control purposes, sitting patterns and usage frequency. Assistance with sitting and standing up and massages	The smart sofa was validated through user testing. (user testing)	4—Limited user testing (no real environment).	No
[25]	2019	Journal Article	Croatia	Force-sensitive resistors (FSR), capacitive sensors, photoplethysmographic (PPG) electrocardiographic (ECG), radar sensors, and flex sensors connected to IoT, Microcontrollers, Arduino, Raspberry Pi, and ESPino32.	Data collection, Process/transmit information, Execute physical/digital actions	Office chairs	Health and well-being, Work/office spaces	Posture Monitoring Health Monitoring. Activity Recognition Posture Correction Assistive Functions Occupancy Detection. Smart chairs combine sensors, IoT and machine learning to promote occupational health in the workplace.	Monitor sitting posture and pressure distribution—provides feedback for correction; heart rate, respiration rate, blood pressure, and blood oxygen levels, user activities based on pressure and movement data.	Validated through user testing and machine learning validation processes. (experimental testing and user testing)	6—Tested in real environment (office).	No
[26]	2017	Conference Paper	Germany	Projector; Camera and Object Recognition; Smartphone Integration, LED Matrix, recorder	Data collection, Process/transmit information, Execute physical/digital actions	Table	Social interaction and communication	Social Interaction Enhancement Media Sharing Object Recognition Remote Communication Recording and Replaying. An interactive table designed to strengthen social connections in smart homes.	Images and other digital media shared by users; information about physical objects placed on the table and their interactions with the system. User Interaction Data, and health Data like heart rate or speed from mobile devices is displayed.	The Prototype was validated through a field study in three households. (user testing)	5—Tested in 3 homes (real scenario, but small sample).	No
[27]	2020	Conference Paper	China	Hetai HT66F2390 microcontroller LCD12864 LCD Display Fingerprint module adopts ATK-AS608 fingerprint recognition module HC-SR501 human body sensing module Bluetooth audio receiver module M38 5 W power amplifier module	Process/transmit information, Execute physical/digital actions	Bedside Table	Efficiency and Daily Life Management	Night light and brightness adjustment refrigeration function heating and insulation device fingerprint unlocking LED light LCD screen. A smart bedside table with multiple integrated functionalities, to optimise space in the bedroom and improve the user experience.	Temperature humidity fingerprint data user interaction data	Used processes to validate the system’s functionalities such as dark environments (to test the nightlight) and with user interactions (such as using the fingerprint module). (experimental testing and user testing)	4—Basic functionalities validated in the laboratory.	No
[28]	2016	Conference Paper	Portugal	Lifting mechanisms (electric, crank, pin slide) LED light sensor Arduino Relay	Process/transmit information, Execute physical/digital actions	Desk	Work/office spaces	Reconfigurable and ergonomic three-level desk	The article does not give attention to data collection, only mentions light intensity data (for automatic lamp activation)	A prototype was built to test the ergonomics and functionality of the table (experimental testing)	6—Ergonomics tests on prototype	Considerations have been made such as: marketing plan, product cost, target market and use of sustainable materials, but it is not yet commercialised
[29]	2023	Conference Paper	Japan	Six-axis sensor (MPU9250) and an ultrasonic sensor (HC-SR04)	Process/transmit information, Execute physical/digital actions	Chair	Efficiency and Daily Life Management	Allows four different ways of sitting, adapting to different user needs and behaviours.	Posture data Load/weight data	Sensor tests (experimental testing)	4—Without testing in real use	No
[30]	2020	Conference Paper	Republic of Korea	Infrared communication sensors and servo motors. Arduino UNO Voice commands	Data collection, Process/transmit information, Execute physical/digital actions	Storage Cabinets	Efficiency and Daily Life Management	Assist users’ organising and finding behaviour	User interaction data: Voice commands User feedback: Perceived usefulness, ease of use, perceived intelligence, product evaluation (via questionnaires).	User testing WOZ technique: Simulated robotic behaviour. Quantitative measurements: Questionnaires (experimental testing and user testing)	5—Tested with users (WOZ technique).	No
[31]	2021	Journal Article	Republic of Korea	Triboelectric Nanogenerator (TENG) Arduino Uno Reactive-Ion Etching (RIE).	Data collection, Process/transmit information, Execute physical/digital actions	Desk	Efficiency and Daily Life Management	Smart desk that triggers an alarm if an intruder accesses it	Electrical output data generated by TENG. Intrusion detection data: Signals triggered by contact with the table.	Electrical performance testing Stability testing Real-time application testing Finite element simulation: COMSOL Multiphysics to validate the working mechanism. (experimental testing)	5—Validated in laboratory and simulations (without external testing).	No
[32]	2016	Journal Article	Finland, Norway, Germany and China	force plates—FP3 infrared sensors camera and facial recognition module Bluetooth pressure sensors	Data collection, Process/transmit information	2 Chairs 1 Mirror	Health and well-being, Targeted to older people	Fall detection and monitoring Older person monitoring and check-in	chairs: quantitative data on physical function (user’s position) mirror: facial data	usability studies with older adults interviews (user testing)	6—Tested in usability studies.	No
[33]	2023	Conference Paper	Italy	Cloud platform Sensors: temperature, humidity, air quality (PM10, PM2.5, PM1, CO, CH4, VOC), atmospheric pressure, brightness, and soil moisture.	Data collection, Process/transmit information	Outdoor furniture (e.g., benches, vases, fountains, street furniture)	Environment monitoring	Monitors and controls garden parameters. Provides automated garden management via IoT. Enhances user experience through interaction with an app.	Air quality, temperature, humidity, pollution levels. Soil moisture, irrigation status. App usage and system control.	Rapid prototyping; Testing of 3D-printed models and manual/digital modifications. Scenario-based design: Application scenarios defined through focus groups and iterative revisions. (experimental testing and user testing)	4—Tested in simulated scenarios.	No
[34]	2023	Conference Paper	Australia	Wireless sensor nodes (Arduino Uno R3, ESP-13 WiFi shield) ultrasonic sensors temperature sensors	Data collection, Process/transmit information	Outdoor furniture—bins, seats, bus shelters	Environment monitoring	Environmental monitoring (temperature, humidity, garbage level, air quality), crowd measurement (via WiFi-enabled devices), and real-time data communication.	Environmental data (temperature, humidity, garbage level, air quality), WiFi probe requests (MAC addresses, signal strengths).	Experiments conducted with five bins deployed, comparing static and dynamic communication models (experimental testing)	5—Tested in an urban environment (small scale).	No
[35]	2024	Conference Paper	Germany	capacitive sensors microcontrollers Bluetooth actuators	Data collection, Execute physical/digital actions	Office chairs	Health and well-being, Work/office spaces	Promotes dynamic sitting posture, reduces muscular tension, and supports active health through haptic feedback.	Sitting posture, pressure distribution, duration in specific positions, and force-displacement measurements.	Experiments with 12 test subjects, force-displacement measurements, FEM optimisation, and sensor testing. (experimental testing)	6—Tested with 12 users (empirical data).	No
[36]	2020	Conference Paper	USA and UK	IoT sensors (temperature, humidity, CO_2_, volatile organic compounds VOC, PM, illuminance, motion, sound level, distance) Raspberry Pi RFID tag PoE (Power over Ethernet)—only in version 1 prototype Machine learning algorithms motorised sit-stand feature	Data collection, Process/transmit information, Execute physical/digital actions	Desk	Health and well-being, Work/office spaces	Personalises indoor environmental conditions (thermal, visual, and acoustic comfort), reduces sedentary time, and improves posture and ergonomics.	Thermal comfort data (temperature, humidity), visual comfort data (illuminance, correlated colour temperature), air quality data (CO_2_, VOC, PM), occupancy state, power consumption, and user preferences.	Ongoing development with iterative versions of the desk (Version 1, 2, and 3), incorporating user feedback and sensor data to refine functionality. (experimental testing and user testing)	6—Iterative development (versions 1–3 with user feedback).	No
[37]	2018	Conference Paper	Republic of Korea	Raspberry Pi Arduino UNO Pressure sensors Convolutional Neural Network (CNN) IoT x-y floater and motor Deep Learning (AI)	Data collection, Process/transmit information, Execute physical/digital actions	shoe cabinet	Efficiency and Daily Life Management	Automatic shoe storage, shoe type and colour classification, and shoe recommendation based on user input (clothing and destination).	Shoe images (type and colour), user input (clothing type and destination), and shoe storage status.	No, only prototyped	3—Conceptual prototype without validation.	No
[38]	2020	Conference Paper	China	Raspberry Pi TFT LCD display infrared frame Baidu Voice Assistant	Data collection, Process/transmit information	Mirror	Environment monitoring, Efficiency and Daily Life Management, Social interaction and communication	Acts as a smart home centre platform, controlling home appliances, displaying real-time information (weather, news, time), monitoring home environments, and enabling remote interaction.	Real-time home environment data, user interaction data (touch, voice), and monitoring data (images, security alerts).	No, only prototyped	4—Patent pending (no large-scale testing).	The project is pending patent applications, but no explicit commercialisation details are provided in the text.
[39]	2017	Conference Paper	Italy	Sensors (environmental, acoustic, presence) RFID WiFi NFC	Data collection, Process/transmit information	Smart urban furnishings (benches, worktops, digital islands)	Environment monitoring	Provides innovative services to citizens, monitors urban environments, and enhances public services through data collection and processing.	Environmental data (pollution, emissions, noise levels), urban dynamics, and user interaction data.	Prototype development and testing (collaboration between academia and industry partners) (experimental testing)	5—Tested in academia-industry collaboration.	The project aims to define a sustainable business plan, but no explicit commercialisation details are provided in the text.
[40]	2018	Conference Paper	Germany	Capacitive proximity sensors machine learning algorithms (C4.5 Decision Tree, kNN, SVM, Naive Bayes, Random Forest).	Data collection, Process/transmit information	Sofa	Health and well-being	Detects proximity and motion of the human body to recognise emotional states (e.g., anxiety, interest, relaxation).	proximity, motion, posture, and movement data.	Study with 15 participants, using both elicited and acted emotions, and machine learning evaluation with leave-one-subject-out cross-validation. (user testing)	5—Tested with 15 participants.	No
[41]	2022	Conference Paper	USA	ultrasonic sensors Xbee 802.15.4 PRO RF Modules Leap Motion controller Arduino stepper motors linear actuators LED	Execute physical/digital actions, Data collection, Process/transmit information	Robotic furniture suite (chair, side table, and screen).	Targeted to older people, Efficiency and Daily Life Management	Assists elderly users with mobility and daily activities, enabling ageing in place.	User gestures, chair occupancy, and table/screen positioning data.	Initial experiments with senior citizens, focusing on usability and functionality. (user testing)	4—Initial usability testing.	No
[42]	2017	Conference Paper	Germany	Textile capacitive sensing electrodes OpenCapSense board (microcontroller)	Data collection, Process/transmit information	Sofa	Health and well-being	Posture detection and recognition	Capacitive sensor data for posture recognition (e.g., sitting upright, leaning back, lying down)	Leave-one-subject-out cross-validation with 15 test persons and 14 postures (user testing)	5—Validated with 15 users (cross-validation).	No
[43]	2015	Conference Paper	Colombia	Proximity and distance sensors ultrasonic sensors Raspberry Pi Zigbee wireless communication traction mechanisms	Data collection, Process/transmit information, Execute physical/digital actions	Chairs and tables	Efficiency and Daily Life Management	Automated spatial configuration of work environments. The furniture pieces move around to adapt to the needs of the occasion.	Distance measurements, spatial positioning data, and user input for configuration settings	No	4—Prototype without testing in a real environment.	No
[44]	2022	Conference Paper	Greece	Weight Sensors PLC (Programmable Logic Controller)	Data collection, Execute physical/digital actions	Main entrance furniture piece	Efficiency and Daily Life Management	Face recognition, reminders, information display, storage for small objects	Face recognition data, user interaction data (e.g., reminders, clothing suggestions), and sensor data for object detection	No	5—Market plan, but without implementation.	The product is intended for both the Greek and European markets, with a profit-loss analysis suggesting potential profitability if launched in both markets. Prices are competitive for high-value furniture, but no specific commercialization details are provided.
[45]	2024	Journal Article	Australia	Sensors Wi-Fi IoT	Data collection, Process/transmit information, Execute physical/digital actions	Hub: Street Furniture	Environment monitoring Social interaction and communication	Create flexible places to meet, work, and play. combat rising urban temperatures. Capture local environmental conditions	Number of users; Environmental Data; Resource Consumption Data: Power usage, water usage; Performance and status of the public furnishings	surveys with both users and non-users to evaluate public perception and usage (user testing)	9—Actual system proven in operational environment	Product already commercialised
[46]	2024	Journal Article	Egypt	IoT, Sensors	Data collection, Process/transmit information, Execute physical/digital actions	Mirror and Drawer	Efficiency and Daily Life Management	Monitor environmental conditions; Collect real-time data on space usage	Real-time user behaviour, environmental conditions, space usage patterns	Conceptual framework; proposed validation via simulation and pilot deployments (experimental testing)	4—Prototype without testing in real scenarios.	No (theoretical framework only)
[47]	2024	Journal Article	Egypt	IoT, Sensors, Arduino, User interfaces, LED	Data collection, Process/transmit information Execute physical/digital actions	Table	Targeted at old people Health and well-being	Surface colour changes to warn users (children, deaf and older people) of hot components that could harm Them.	Surface temperature, presence of thermal objects, and thermal risk level	Lab Testing (experimental testing)	4—Prototype without testing in real scenarios.	No
